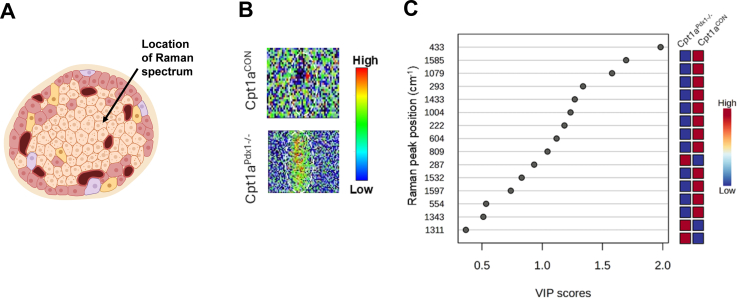# Pancreatic expression of CPT1A is essential for whole body glucose homeostasis by supporting glucose-stimulated insulin secretion

**DOI:** 10.1016/j.jbc.2025.108187

**Published:** 2025-01-13

**Authors:** Maggie P. Ducote, Caroline R. Cothern, Heidi M. Batdorf, Molly S. Fontenot, Thomas M. Martin, Maria Iftesum, Manas R. Gartia, Robert C. Noland, David H. Burk, Sujoy Ghosh, Susan J. Burke

**Affiliations:** 1 Laboratory of Immunogenetics, Pennington Biomedical Research Center, Baton Rouge, Louisiana, USA; 2 Laboratory of Islet Biology and Inflammation, Pennington Biomedical Research Center, Baton Rouge, Louisiana, USA; 3 Department of Biological Sciences, Louisiana State University, Baton Rouge, Louisiana, USA; 4 Department of Mechanical and Industrial Engineering, Louisiana State University, Baton Rouge, Louisiana, USA; 5 Skeletal Muscle Metabolism Laboratory, Pennington Biomedical Research Center, Baton Rouge, Louisiana, USA; 6 Cell Biology and Bioimaging Core, Pennington Biomedical Research Center, Baton Rouge, Louisiana, USA; 7 Laboratory of Computational Biology, Pennington Biomedical Research Center, Baton Rouge, Louisiana, USA

**Keywords:** CPT1a activity, pancreatic triglyceride, β-cell dedifferentiation, glucose homeostasis, pancreatic islet β-cell function

## Abstract

Pancreatic islet β-cells express the *Cpt1a* gene, which encodes the enzyme carnitine palmitoyltransferase 1A (CPT1A), an enzyme that facilitates entry of long-chain fatty acids into the mitochondria. Because fatty acids are required for glucose-stimulated insulin secretion, we tested the hypothesis that CPT1A is essential to support islet β-cell function and mass. In this study, we describe genetic deletion of *Cpt1a* in pancreatic tissue (Cpt1a^**Pdx1−/−**^) using C57BL/6J mice. Islet morphology, β-cell transcription factor abundance, islet ATP levels, glucose transporter 2 abundance, and expression of the dedifferentiation marker ALDH1A3 were analyzed by immunofluorescent staining. Glucose and insulin tolerance were assessed to investigate the metabolic status of genetic reductions in *Cpt1a*. Glucose-stimulated insulin secretion was evaluated *in vivo* and in isolated islets *ex vivo* by perifusion. Pancreatic deletion of *Cpt1a* reduced glucose tolerance but did not alter insulin sensitivity. Glucose-stimulated insulin secretion was reduced both *in vivo* and in islets isolated from Cpt1a^**Pdx1−/−**^ mice relative to control islets. Pancreatic islets from Cpt1a^**Pdx1−/−**^ mice displayed elevations in ALDH1A3, a marker of dedifferentiation, but no reduction in nuclear abundance of the β-cell transcription factors MafA and Nkx6.1 or the GLUT2 glucose transporter. However, intracellular ATP abundance was markedly decreased in islets isolated from Cpt1a^**Pdx1−/−**^ relative to littermate control mice. We conclude that there is an important physiological role for pancreatic CPT1A to maintain whole body glucose homeostasis by supporting glucose-stimulated insulin secretion and maintaining intracellular ATP levels in male mice.

Pancreatic islet β-cells respond to nutritional stimuli with regulated insulin secretion. Perhaps the best-known stimulus is glucose, where metabolism of the carbohydrate is coupled to closure of K_ATP_ channels and opening of voltage-gated calcium channels. Consequently, changes in circulating glucose levels are sensed and responded to with regulated insulin secretion. Other nutrients, such as lipids, potentiate glucose-stimulated insulin secretion (GSIS) (1).

In addition, the presence of fatty acids supports GSIS. When rats were fasted to deplete tissue fatty acids, or if lipolysis was attenuated to restrict circulating fatty acids, GSIS was markedly reduced (2). In addition, in the presence of experimental hyperleptinemia, which reduces the tissue lipid content to near zero, the β-cells are also unresponsive to glucose as a secretory signal (3). On the other hand, if lipid accumulates to pathologic levels within the pancreatic tissue, GSIS is impaired (4). Thus, the islet β-cell has a tightly regulated relationship with fatty acids to appropriately regulate GSIS.

In this study, we generated the first *in vivo* model of pancreatic reduction in mitochondrial lipid entry by using genetic deletion of *Cpt1a*. The carnitine palmitoyltransferase group of enzymes is encoded by the *Cpt1a*, *Cpt1b*, and *Cpt1c* genes. CPT1A is enriched in pancreatic tissue, including in islet β-cells. CPT1A is an enzyme that catalyzes a rate-limiting step of fatty acid transport into mitochondria by transferring carnitine to acyl-CoAs. This key intracellular chemical reaction allows long-chain fatty acids to enter the mitochondrial matrix for oxidation, generating large amounts of ATP (5). By producing a novel genetic model with pancreatic deletion of *Cpt1a*, we were able to test the hypothesis that entry of long-chain fatty acids into the mitochondria is critical to support islet β-cell function *in vivo* and *ex vivo*, and overall glucose homeostasis *in vivo*.

In contrast to previous studies performed using *in vitro* model systems (*e.g.,* perfused pancreata and insulinoma cell lines) that showed an *enhancement* of GSIS upon inhibition of CPT1 activity (6, 7, 8), we found that male mice with *Cpt1a* deletion in both exocrine and endocrine tissue were glucose intolerant with *reduced* GSIS. RNA-seq of pancreatic islets yielded very few changes at the transcriptional level in response to deletion of *Cpt1a*. By contrast, islet ATP levels were greatly reduced, consistent with a role for fatty acid metabolism to provide key signals supporting islet β-cell function *in vivo* and *ex vivo*. We conclude that earlier *in vivo* studies showing that circulating fatty acids were necessary for β-cell function were accurate and that our data provide a mechanism for these effects, which is metabolism of fatty acids to support islet ATP levels.

## Results

### Loss of CPT1A function in the pancreas of male mice promotes increased pancreatic and islet triglyceride (TG) accumulation with no change in body composition, energy expenditure, respiratory quotient, or insulin sensitivity

**Figure 1:**
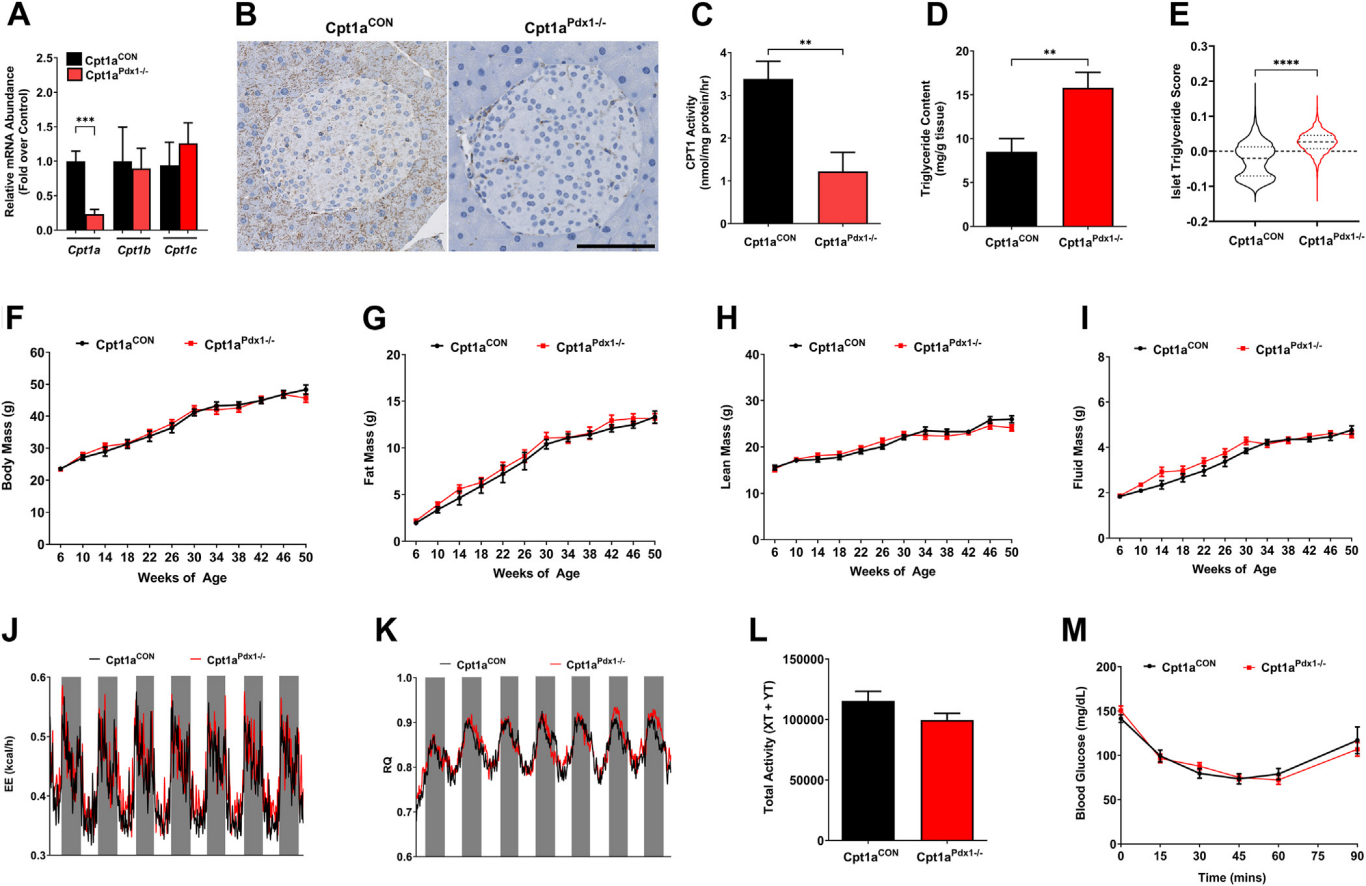
**Loss of CPT1A function in the pancreas of male mice promotes increased pancreatic and islet triglyceride accumulation with no change in body composition, energy expenditure, respiratory quotient, or insulin sensitivity.***A*, expression of *Cpt1a, Cpt1b,* and *Cpt1c* were measured by RT-PCR using RNA isolated from islets, (*B*) CPT1A abundance in formalin-fixed pancreatic tissue, (*C*) CPT1 enzymatic activity in mitochondrial suspensions isolated from whole pancreatic tissue, (*D*) triglyceride content measured from whole pancreatic tissue, (*E*) islet triglyceride scores, (*F*) body mass, (*G*) fat mass, (*H*), lean mass, (*I*) fluid mass, (*J*) energy expenditure (EE) across the light (*white bars*) and dark (*gray bars*) cycle over a 7 day period, (*K*) respiratory quotient (RQ) across the light (*white bars*) and dark (*gray bars*) cycle over a 7 day period, (*L*) total activity quantified by the total number of X and Y beam breaks (XT + XY) over a 7 day period, and (*M*) an insulin tolerance test in 20 week old mice. All experiments were performed in male littermate Cpt1a^CON^*versus* Cpt1a^Pdx1−/−^ mice. n = 8 to 10 per group (*A*–*D* and *F*–*M*), n = 4 (*E*). A representative islet in (*B*) was chosen from each genotype. ∗∗*p* < 0.01, ∗∗∗*p* < 0.001, and ∗∗∗∗*p* < 0.0001. CPT1A, carnitine palmitoyltransferase 1A.

Circulating fatty acids are critical for insulin secretion (2). Since CPT1A is rate limiting for long-chain fatty acid metabolism, we tested the hypothesis that CPT1A activity in the pancreas regulates islet β-cell function. To generate a mouse model with a pancreas-targeted deletion, mice with conditional “floxed” alleles were crossed to mice expressing Cre recombinase under the control of the Pdx1 promoter (Cpt1a^Pdx1−/−^). Expression of the *Cpt1a* gene decreased 75.7% in islets isolated from Cpt1a^Pdx1−/−^ mice compared to littermate controls (Fig. 1*A*). Confirming that our mouse model is specific for the *Cpt1a* isoform, we observed no loss in *Cpt1b* or *Cpt1c* mRNA expression in islets from Cpt1a^Pdx1−/−^ mice compared to controls (Fig. 1*A*). Staining of formalin-fixed paraffin-embedded (FFPE) pancreatic tissue showed that CPT1A is expressed in both endocrine and exocrine compartments of the pancreas of Cpt1a^CON^ mice, but is almost completely absent in the pancreatic tissue, including pancreatic islets, of Cpt1a^Pdx1−/−^ mice (Fig. 1*B*). CPT1 enzymatic activity was reduced by 65% in pancreatic mitochondria of Cpt1a^Pdx1−/−^ compared to littermate controls (Fig. 1*C*). Residual CPT1A protein abundance and enzymatic activity detected in Cpt1a^Pdx1−/−^ mice is likely due to the presence of cells that do not express Pdx1 (*e.g.,* fibroblasts, macrophages, endothelial cells, *etc.*). The CPT1A enzyme plays a critical role in partitioning of fatty acid metabolism toward mitochondrial oxidation. Therefore, we hypothesized that loss of CPT1 activity in the pancreas would promote accrual of excess lipid. As expected, TG content was increased 1.86-fold in pancreatic tissue of Cpt1a^Pdx1−/−^ mice compared to controls (Fig. 1*D*), consistent with observed reductions in CPT1 enzymatic activity. Based on analysis of 6494 and 3965 Raman spectra from islets of Cpt1a^CON^ and Cpt1a^Pdx1−/−^ mice, respectively, the relative TG score was found to be higher in islets from Cpt1a^Pdx1−/−^ mice than Cpt1a^CON^ controls (Fig. 1*E*).

**Supplementary Figure 1:**
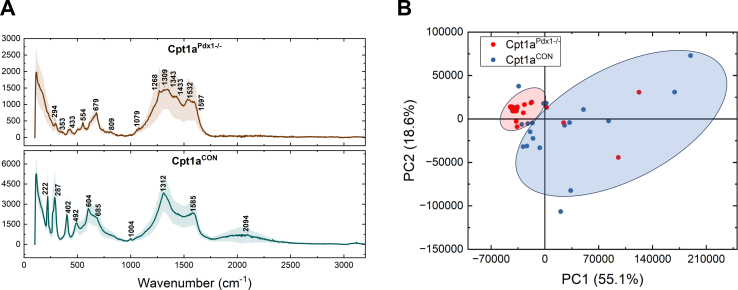


Further, Raman spectra analysis identified increased intensity of several Raman peaks at 809, 1309, and 1343 cm⁻^1^ in the islets from Cpt1a^Pdx1−/−^ mice compared to the control islets (Figs. S1 and S2). A Raman peak at 809 cm^−1^ is often attributed to phospholipid vibrations, such as P-O-C stretching or symmetric phosphate group vibrations in phospholipids and nucleic acids. Increased intensity may reflect an accumulation of membrane phospholipids. The peak at 1309 cm^−1^ is often associated with CH2 twisting and wagging vibrations in fatty acids and lipids, particularly in saturated lipid chains, and the Raman peak at 1343 cm^−1^ is associated with CH3 and CH2 bending vibrations in lipids, proteins, and potentially DNA.

Between the ages of 6 and 50 weeks, there were no obvious changes in body mass (Fig. 1*F*), fat mass (Fig. 1*G*), lean mass (Fig. 1*H*), or fluid mass (Fig. 1*I*), in male Cpt1a^Pdx1−/−^ mice compared to littermate controls. Similarly, other whole body parameters including energy expenditure (Fig. 1*J*), respiratory quotient (Fig. 1*K*), spontaneous locomotor activity (Fig. 1*L*), and peripheral insulin sensitivity (Fig. 1*M*) were not impacted by pancreatic deletion of the CPT1A protein in male Cpt1a^Pdx1−/−^ mice.

### Decreased *Cpt1a* expression in the pancreas of female mice correlates with increased pancreatic TG accrual but no change in insulin sensitivity

**Figure 2:**
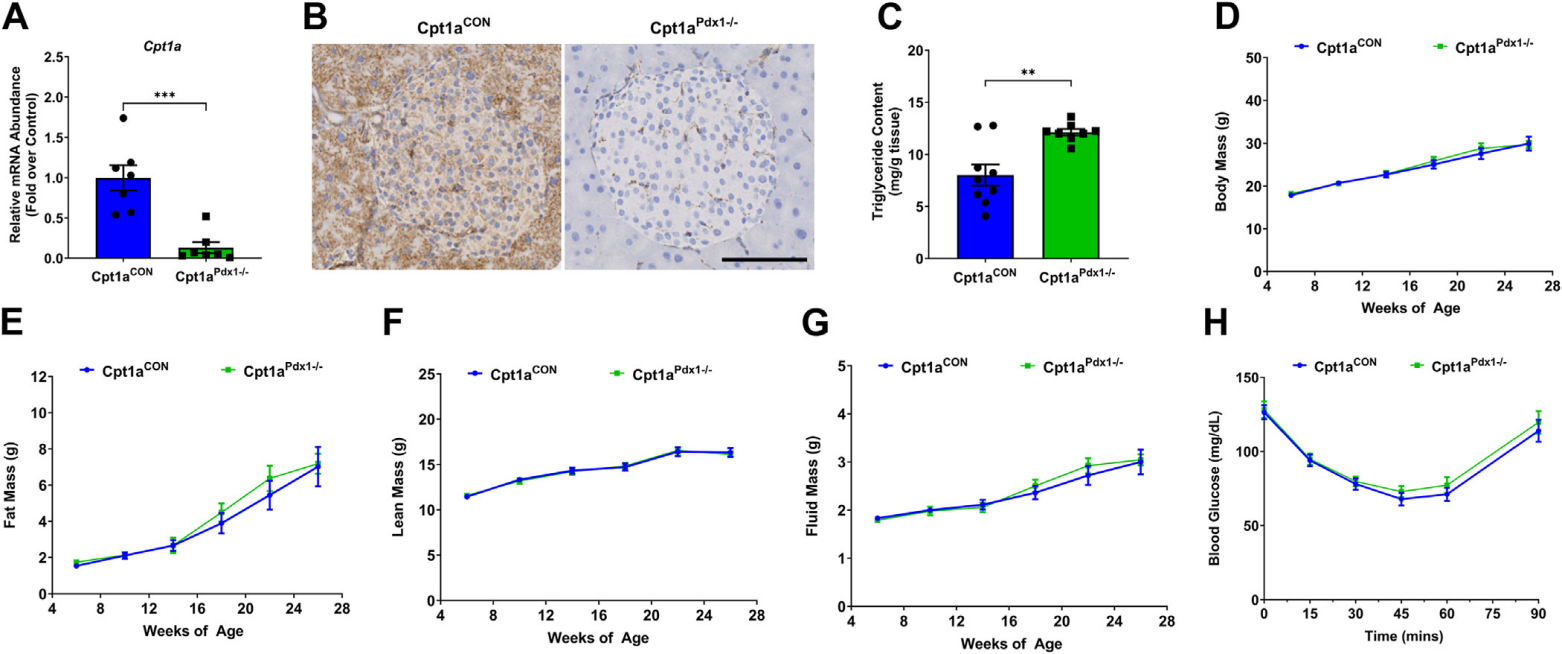
**Decreased *Cpt1a* expression in the pancreas of female mice correlates with increased pancreatic triglyceride accrual but no change in insulin sensitivity.***A*, expression of *Cpt1a* was measured by RT-PCR using RNA isolated from islets, (*B*) CPT1A abundance in formalin-fixed pancreatic tissue, (*C*) triglyceride content from whole pancreatic tissue, (*D*) body mass, (*E*) fat mass, (*F*), lean mass, (*G*) fluid mass, (*H*) an insulin tolerance test in 25 week old mice. All experiments were performed in female littermate Cpt1a^CON^*versus* Cpt1a^Pdx1−/−^ mice (n = 9–10 per group). A representative islet in (*B*) was chosen from each genotype. ∗∗*p* < 0.01 and ∗∗∗*p* < 0.001. CPT1A, carnitine palmitoyltransferase 1A.

Female Cpt1a^Pdx1−/−^ mice have a 96.9% reduction in islet *Cpt1a* gene expression (Fig. 2*A*). CPT1A abundance is almost undetectable by immunohistochemical (IHC) analysis in both exocrine and endocrine compartments of female Cpt1a^Pdx1−/−^ mice compared to littermate controls (Fig. 2*B*). Pancreatic TG content is significantly increased (1.51-fold) compared to Cpt1a^CON^ mice (Fig. 2*C*). Like male mice, loss of pancreatic *Cpt1a* abundance in female Cpt1a^Pdx1−/−^ mice does not alter body composition (Fig. 2, *D*–*G*) or insulin sensitivity (Fig. 2*H*).

### Deletion of CPT1A in pancreatic tissue reduces glucose tolerance and **β**-cell function in male mice, but the ability of **β**-cells to proliferate in response to metabolic adaptations is retained

**Figure 3:**
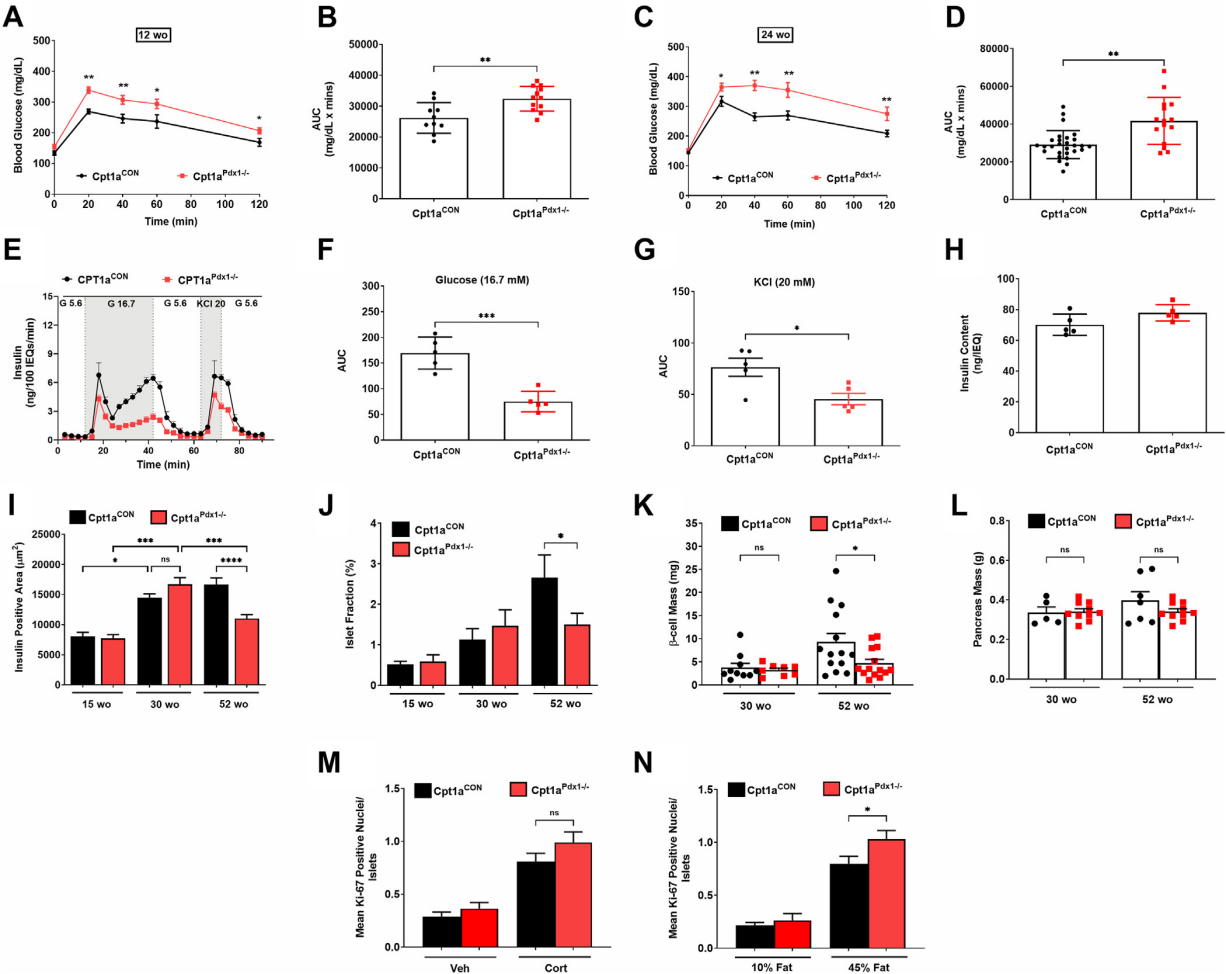
**Deletion of CPT1A in pancreatic tissue reduces glucose tolerance and β-cell function in male mice, but the ability of β-cells to proliferate in response to metabolic adaptations is retained.** Glucose tolerance tests in 12 week old (*A*) and 24 week old (*C*) male Cpt1a^CON^*versus* Cpt1a^Pdx1−/−^ mice with respective area under the curve (AUC) calculations (*B*, *D*). *E*, glucose- and KCl-stimulated insulin secretion in islets isolated from 12 week old male Cpt1a^CON^*versus* Cpt1a^Pdx1−/−^ mice with respective AUC calculations for glucose- (*F*) and KCl-stimulated time points (*G*), that is, time points highlighted in *gray* in *panel E*. *H*, insulin content in the same islets as shown in *panel E*. *I*, insulin positive area and (*J*) islet fraction calculated from formalin-fixed pancreatic tissue in 15 week old, 30 week old, and 52 week old male Cpt1a^CON^*versus* Cpt1a^Pdx1−/−^ mice. *K*, β-cell mass and (*L*) pancreas mass in 30 week old and 52 week old male Cpt1a^CON^*versus* Cpt1a^Pdx1−/−^ mice. Quantification of the number of Ki-67^+^ nuclei per islet in 30 week old male Cpt1a^CON^*versus* Cpt1a^Pdx1−/−^ mice treated with Vehicle or Cort for 1 week (*M*) or following a 1 week exposure to 10% or 45% fat purified diet (*N*). n = 10 to 12 (*A*, *B*), n = 15 to 28 (*C*, *D*), n = 5 (*E*–*H*), n = 14 (*I*–*L*), n = 10 (*M*, *N*); ns = not significant, ∗*p* < 0.05, ∗∗*p* < 0.01, ∗∗∗*p* < 0.001, and ∗∗∗∗*p* < 0.0001. CPT1A, carnitine palmitoyltransferase 1A.

Deletion of CPT1A in pancreatic tissue and subsequent increases in pancreatic and islet TG content (Fig. 1) correlated with a decline in glucose tolerance in male Cpt1a^Pdx1−/−^ mice. Between 6 and 8 weeks of age, glucose intolerance was modestly, but not significantly, increased (data not shown) in male Cpt1a^Pdx1−/−^ mice compared to littermate controls. Glucose tolerance was significantly impaired in male Cpt1a^Pdx1−/−^ mice by 12 weeks of age (Fig. 3, *A* and *B*), 24 weeks of age (Fig. 3, *C* and *D*), and maintained until our last measurement at 52 weeks of age (data not shown). Heterozygous Cpt1a^Pdx1+/−^ mice displayed no impairment in glucose tolerance between 12 and 24 weeks of age (data not shown). As a likely explanation for impaired glucose tolerance in Cpt1a^Pdx1−/−^ mice, we observed a decline in glucose- and KCl-induced insulin secretion in isolated islets (Fig. 3, *E*–*G*) with no change in insulin content in the same islets (Fig. 3*H*). Insulin positive area increased in both control and Cpt1a^Pdx1−/−^ mice at 30 weeks of age relative to 15 weeks of age (Fig. 3*I*). However, by 52 weeks of age, there was a clear decrease in insulin-positive area (Fig. 3*I*), islet fraction in the pancreas (Fig. 3*J*), and β-cell mass (Fig. 3*K*), despite no change in overall pancreatic mass (Fig. 3*L*) in the Cpt1a^Pdx1−/−^ mice relative to littermate control mice. Importantly, decreased insulin secretion in 12 week old male Cpt1a^Pdx1−/−^ mice is not associated with a decrease in islet insulin content (Fig. 3*H*), insulin-positive area (Fig. 3*I*), or β-cell mass (Fig. 3*K*).

We next exposed the mice to discrete challenges that promote islet β-cell proliferation, namely acute corticosterone exposure (9) and high-fat diet (HFD) (10). In both situations, the Cpt1a^Pdx1−/−^ mice showed largely normal phenotypic responses (Fig. 3*M*; Cort) or displayed an increase in Ki-67+ nuclei in β-cells costaining with insulin (Fig. 3*N*; HFD). We conclude that preventing the ability of β-cells to metabolize long-chain fats through CPT1A does not restrict their ability to respond to a signal that triggers proliferation.

### CPT1a activity is required for the lipid-mediated potentiation of GSIS, and activity is not altered by dietary fat content

**Figure 4:**
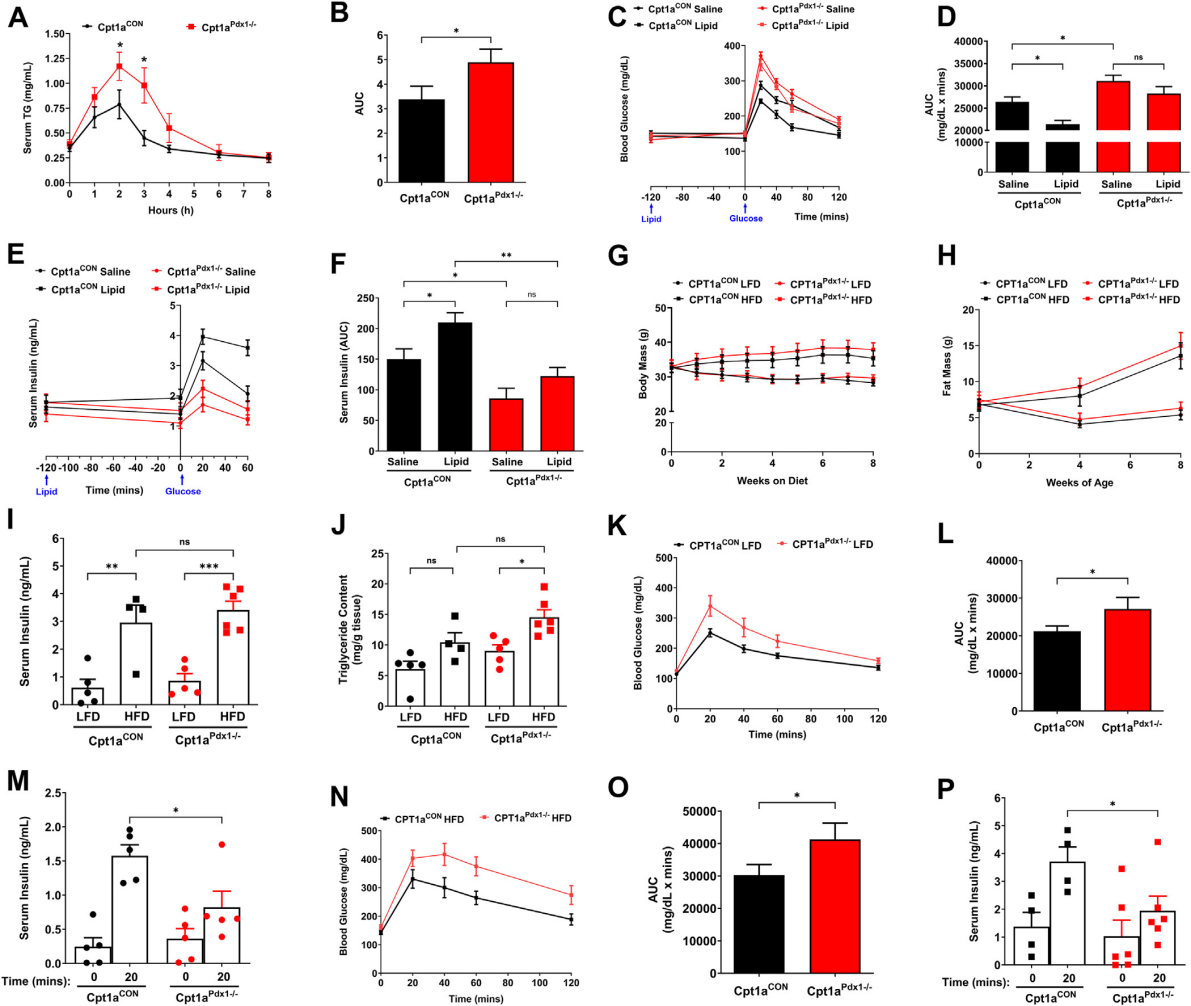
**CPT1a activity is required for the lipid-mediated potentiation of GSIS, and activity is not altered by dietary fat content.***A* and *B*, serum triglycerides (TGs) were assessed following oral gavage of a 20% intralipid emulsion in 15 week old male Cpt1a^CON^*versus* Cpt1a^Pdx1−/−^ mice. *C* and *D*, glucose tolerance and (*E*, *F*) serum insulin were determined in 15 week old male Cpt1a^CON^*versus* Cpt1a^Pdx1−/−^ mice given an oral gavage of saline or 20% intralipid emulsion at −120 min, followed by intraperitoneal injection of glucose at 0 min. Time points of lipid and glucose administration are indicated by *blue arrows*. *G*, body mass, (*H*) fat mass, (*I*) serum insulin, and (*J*) pancreatic triglyceride content were assessed in male Cpt1a^CON^*versus* Cpt1a^Pdx1−/−^ mice on low- or high-fat diet for 8 weeks. *K* and *L*, glucose tolerance and (*M*) *in vivo* GSIS were assessed in male Cpt1a^CON^*versus* Cpt1a^Pdx1−/−^ mice on low-fat diet for 8 weeks. *N* and *O*, glucose tolerance and (*P*) *in vivo* GSIS were assessed in male Cpt1a^CON^*versus* Cpt1a^Pdx1−/−^ mice on high-fat diet for 8 weeks. n = 10 to 14 (*A*–*F*), n = 4 to 6 (*G*–*P*). ns = not significant, ∗*p* < 0.05, ∗∗*p* < 0.01, ∗∗∗*p* < 0.001. LFD, low fat diet, HFD, high-fat diet; CPT1A, carnitine palmitoyltransferase 1A; GSIS, glucose-stimulated insulin secretion.

Acute increases in fatty acid availability can potentiate GSIS; however, the mechanisms have only been examined *in vitro* (2, 11). Infusion of intralipid in the absence of glucose is not sufficient to promote release of insulin in rats (2) or normoglycemic humans during hyperglycemic clamps (11). However, in the presence of glucose, lipid potentiates insulin secretion in both humans and rats. We hypothesized that this lipid potentiation of GSIS requires CPT1a activity. First, we showed that systemic administration of a 20% intralipid emulsion, *via* oral gavage, promotes acute hypertriglyceridemia that peaks at 2 h in male Cpt1a^CON^ mice and is cleared systemically by 3 h. The clearance of lipid in serum is slower in Cpt1a^Pdx1−/−^ mice, with serum TGs returning to baseline levels by 4 h (Fig. 4, *A*–*B*). Next, animals were administered with either saline or 20% intralipid 2 h prior to delivery of a glucose bolus. In saline-treated animals, we see impaired glucose tolerance in Cpt1a^Pdx1−/−^ mice compared to Cpt1a^CON^ mice (Fig. 4, *C* and *D*). These data are consistent with a decrease in glucose-dependent insulin secretion (Fig. 4, *E* and *F*). Lipid treatment in Cpt1a^CON^ mice promoted a smaller rise in blood glucose concentrations with faster clearance than saline treated Cpt1a^CON^ mice (Fig. 4, *C* and *D*), correlating with a higher and more prolonged excursion in insulin (Fig. 4, *E* and *F*). Most notably, lipid treatment in Cpt1a^Pdx1−/−^ mice promoted an almost identical rise in blood glucose as seen in saline-treated Cpt1a^Pdx1−/−^ mice in response to i.p. glucose administration (Fig. 4, *C* and *D*), and GSIS was not significantly different between these groups (Fig. 4, *E* and *F*). These data confirm that there is a clear dependence on CPT1a activity for the lipid potentiation of GSIS *in vivo*.

Reductions in glucose tolerance and β-cell function are observed in male mice lacking pancreatic CPT1a activity (Fig. 3). To determine whether modulating the dietary fat content could alter these phenotypes, we performed an 8-week dietary intervention in male Cpt1a^CON^ and Cpt1a^Pdx1−/−^ mice. Starting at 24 weeks of age, when glucose tolerance and GSIS are impaired in animals on standard chow diet (Fig. 3, *A*–*G*), but prior to loss of β-cell mass (Fig. 3*K*), animals were randomly assigned to either a 13% (LFD; low-fat diet) or 44.3% kcal from fat (HFD) nonpurified chow diet. Glucose tolerance was also assessed in this cohort prior to beginning the dietary intervention and results were as previously shown in Figure 3, *C* and *D* (data not shown). Over the course of 8 weeks, no difference was observed in body mass (Fig. 4*G*), fat mass (Fig. 4*H*), or random-fed blood glucose (data not shown) between the two genotypes; differences observed were only attributed to dietary fat intake. At the completion of the 8-week dietary intervention, HFD-induced hyperinsulinemia was present in both Cpt1a^CON^ in Cpt1a^Pdx1−/−^ mice, with no statistical differences between the genotypes (Fig. 4*I*). HFD also promoted a significant increase in pancreatic TG content in Cpt1a^Pdx1−/−^, but not Cpt1a^CON^ mice, however, due to an upward trend in the HFD-treated Cpt1a^CON^ mice, there is no statistical difference between the genotypes (Fig. 4*J*). Glucose tolerance was significantly increased in Cpt1a^Pdx1−/−^ mice on both the LFD (Fig. 4, *K* and *L*) and HFD (Fig. 4, *N* and *O*) compared to controls. *In vivo* GSIS was impaired in Cpt1a^Pdx1−/−^ mice on both LFD (Fig. 4*M*) and HFD (Fig. 4*P*). Thus, CPT1a activity is required for maintenance of glucose tolerance and GSIS regardless of dietary fat intake.

### CPT1A activity is dispensable for maintenance of **β**-cell function and glucose tolerance in female mice

**Figure 5:**
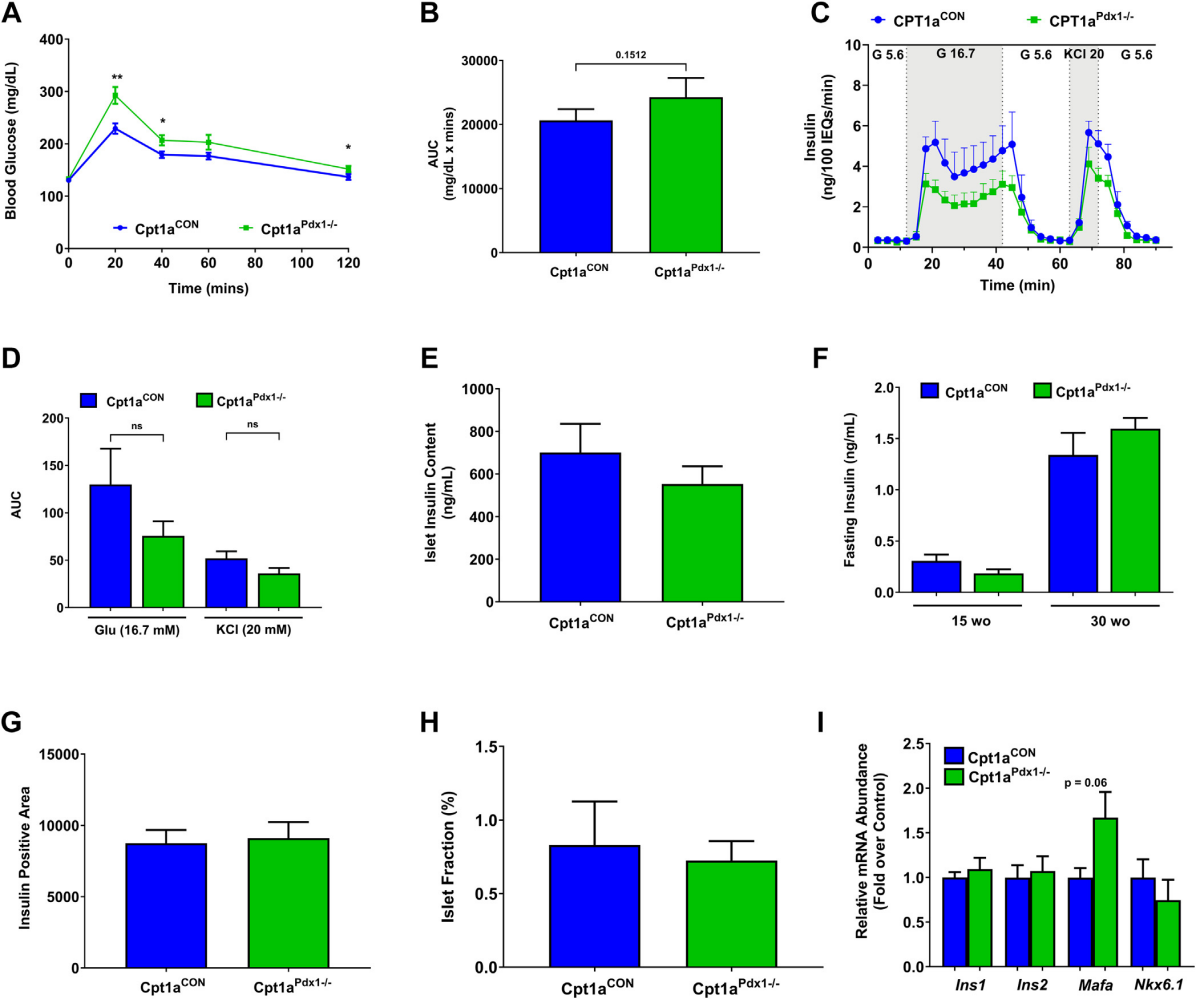
**CPT1A activity is dispensable for maintenance of β-cell function and glucose tolerance in female mice.***A*, A glucose tolerance test in 16 to 28 week old female Cpt1a^CON^*versus* Cpt1a^Pdx1−/−^ mice with respective AUC calculation (*B*). *C*, glucose- and KCl-stimulated insulin secretion in islets isolated from 30 week old female Cpt1a^CON^*versus* Cpt1a^Pdx1−/−^ mice with respective AUC calculations for glucose- and KCl-stimulated time points (*D*), that is, time points highlighted in *gray* in *panel C*. *E*, insulin content in the same islets as shown in *panel C*. *F*, fasting serum insulin in 15 week old and 30 week old female Cpt1a^CON^*versus* Cpt1a^Pdx1−/−^ mice. *G*, insulin-positive area and (*H*) islet fraction calculated from formalin-fixed paraffin-embedded pancreatic tissue in 30 week old female Cpt1a^CON^*versus* Cpt1a^Pdx1−/−^ mice. *I*, *Ins1, Ins2, Mafa,* and *Nkx6.1* mRNA levels in isolated islets from 30 week old female Cpt1a^CON^*versus* Cpt1a^Pdx1−/−^ mice. n = 23 to 28 (*A* and *B*), n = 3 to 5 (*C*–*E*), n = 7 (*F*–*I*). ns = not significant, ∗*p* < 0.05 and ∗∗*p* < 0.01. CPT1A, carnitine palmitoyltransferase 1A; AUC, area under the curve.

Despite decreased CPT1A abundance and accumulation of excess intrapancreatic TG content in female Cpt1a^Pdx1−/−^ mice, these animals only display minor impairments in glucose tolerance (Fig. 5, *A* and *B*). Glucose- and KCl-induced insulin secretion in isolated islets is also largely normal (Fig. 5, *C* and *D*). Further, no alteration in islet insulin content (Fig. 5*E*), fasting serum insulin (Fig. 5*F*), insulin-positive area (Fig. 5*G*), islet fraction (Fig. 5*H*), or β-cell transcription factors *Mafa* and *Nkx6.1* were present in female mice with a reduction in pancreatic CPT1A abundance (Fig. 5*I*). Expression of the *Ins1* and *Ins2* genes were also unchanged (Fig. 5*I*).

### Markers of **β**-cell maturity are unchanged in male mice with a pancreatic reduction in CPT1A activity

**Figure 6:**
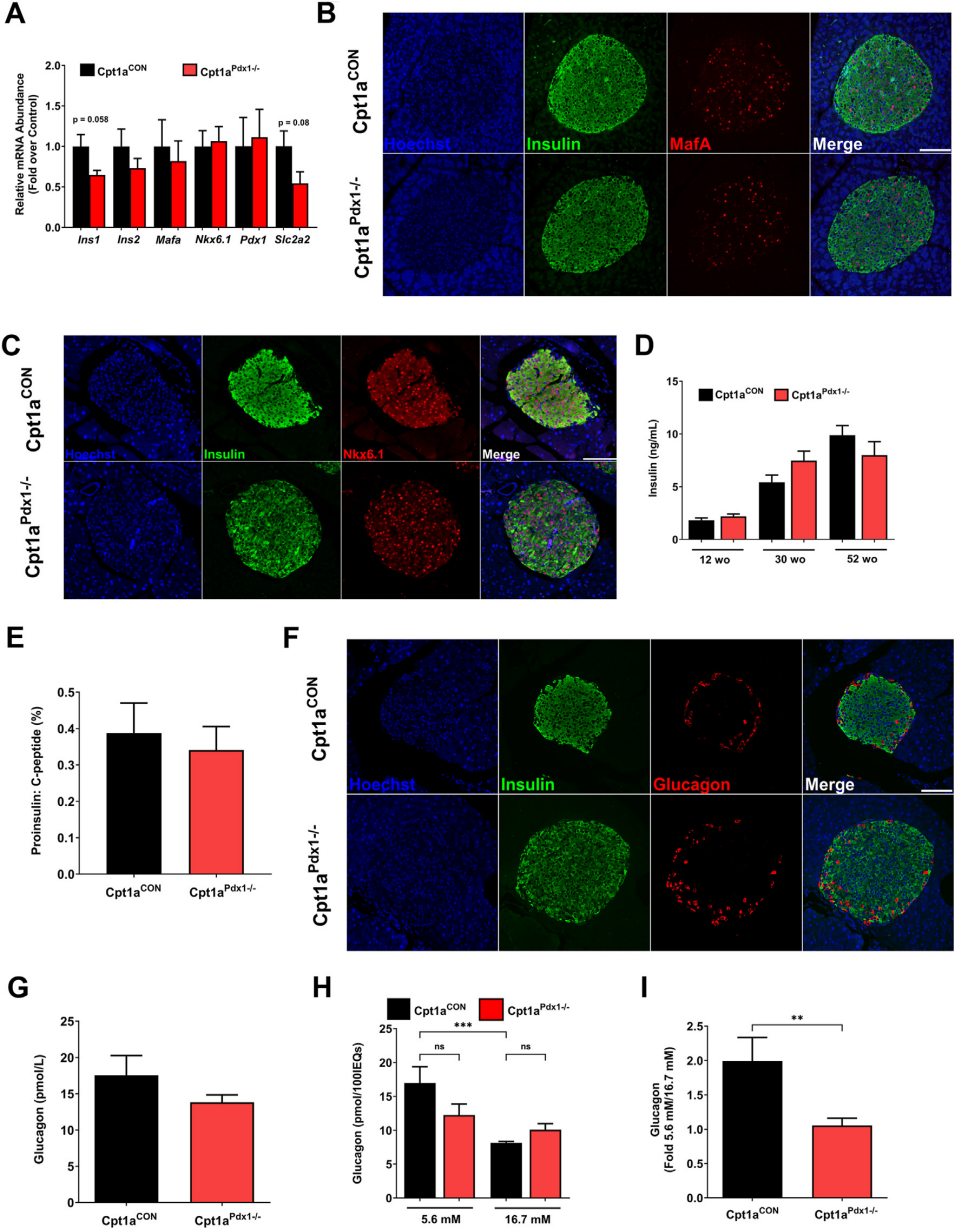
**Markers of β-cell maturity are unchanged in male mice with a pancreatic reduction in CPT1A activity.***A*, *Ins1, Ins2, Mafa, Nkx6.1, Pdx1,* and *Slc2a2* mRNA levels in isolated islets from 30 week male Cpt1a^CON^*versus* Cpt1a^Pdx1−/−^ mice. *B*, immunofluorescence staining for insulin (*green*) costaining with MafA (*red*), Nkx6.1 (*red*; *C*), and glucagon (*red*; *F*) in formalin-fixed paraffin-embedded (FFPE) pancreatic tissue from 30 week old male Cpt1a^CON^*versus* Cpt1a^Pdx1−/−^ mice. *D*, fasting serum insulin in 12 week old, 30 week old, and 52 week old, (*E*) fasting serum proinsulin:C-peptide ratio, and (*G*) fasting serum glucagon in 30 week old male Cpt1a^CON^*versus* Cpt1a^Pdx1−/−^ mice. *H*, glucose-stimulated glucagon secretion, and (*I*) fold change in glucose-stimulated glucagon secretion, in islets isolated from 12 week old male Cpt1a^CON^*versus* Cpt1a^Pdx1−/−^ mice. n = 7 to 8 (*A*), n = 5; the scale bar represents 100 μm (*B*, *C*, and *F*), n = 14 to 24 (*D*), n = 6 (*E*), n = 10 to 15 (*G*), and n = 4 (*H* and *I*). ns = not significant, ∗∗*p* < 0.01, ∗∗∗*p* < 0.001. CPT1A, carnitine palmitoyltransferase 1A.

To establish how deletion of pancreatic CPT1A protein promotes impaired GSIS in male Cpt1a^Pdx1−/−^ mice, we examined several factors related to insulin production, processing, and glucose metabolism. No significant difference was seen between genotypes for either *Ins1* or *Ins2* transcripts, or mRNA levels for the β-cell identity markers, *Mafa*, *Nkx6.1*, or *Pdx1* (Fig. 6*A*). In addition, transcripts for *Slc2a2*, which encodes the glucose transporter 2 (GLUT2), trended lower in Cpt1a^Pdx1−/−^ mice but were not significantly different (Fig. 6*A*). Costaining with insulin confirmed that islet abundance of MafA and Nkx6.1 proteins are not altered in Cpt1a^Pdx1−/−^ mice compared to controls (Fig. 6, *B* and *C*). Basal insulin levels across the lifespan are also unchanged (Fig. 6*D*), and no alteration was observed in the proinsulin: C-peptide ratio between genotypes (Fig. 6*E*).

Glucagon-positive α-cells were not found to be more abundant Cpt1a^Pdx1−/−^ mice (Fig. 6*F*). Additionally, we found that Cpt1a^Pdx1−/−^ mice did not have increased circulating glucagon (Fig. 6*G*). CPT1A is known to contribute to glucagon secretion from pancreatic islets (12); therefore, we also evaluated glucagon secretion in islets isolated from Cpt1a^CON^ and Cpt1a^Pdx1−/−^ mice. A significant decrease in glucagon secretion was observed in Cpt1a^CON^ islets when islets were switched from 5.6 mM to 16.7 mM glucose (Fig. 6*H*). The decreased secretion in glucose in response to 16.7 mM glucose was not observed in islets from Cpt1a^Pdx1−/−^ mice, due to the lower baseline secretion in response to 5.6 mM glucose (Fig. 6*H*). Additionally, the fold change in glucagon secretion from low to high glucose was lower in islets from Cpt1a^Pdx1−/−^ mice than controls (Fig. 6*I*). Overall, we conclude that Cpt1a^Pdx1−/−^ mice have several features consistent with eventual development of type 2 diabetes (T2D) *e.g.,* impaired glucose tolerance and increased pancreatic TGs, but no change in islet glucagon abundance or markers of β-cell maturity (*e.g.*, MafA, Pdx1, Nkx6.1), as noted in models of established disease (*e.g.*, *db/db* mice) (13).

### GLUT2 abundance is unchanged, but the dedifferentiation marker, ALDH1A3, is elevated in **β**-cells from male, but not female, mice deficient in CPT1A

**Figure 7:**
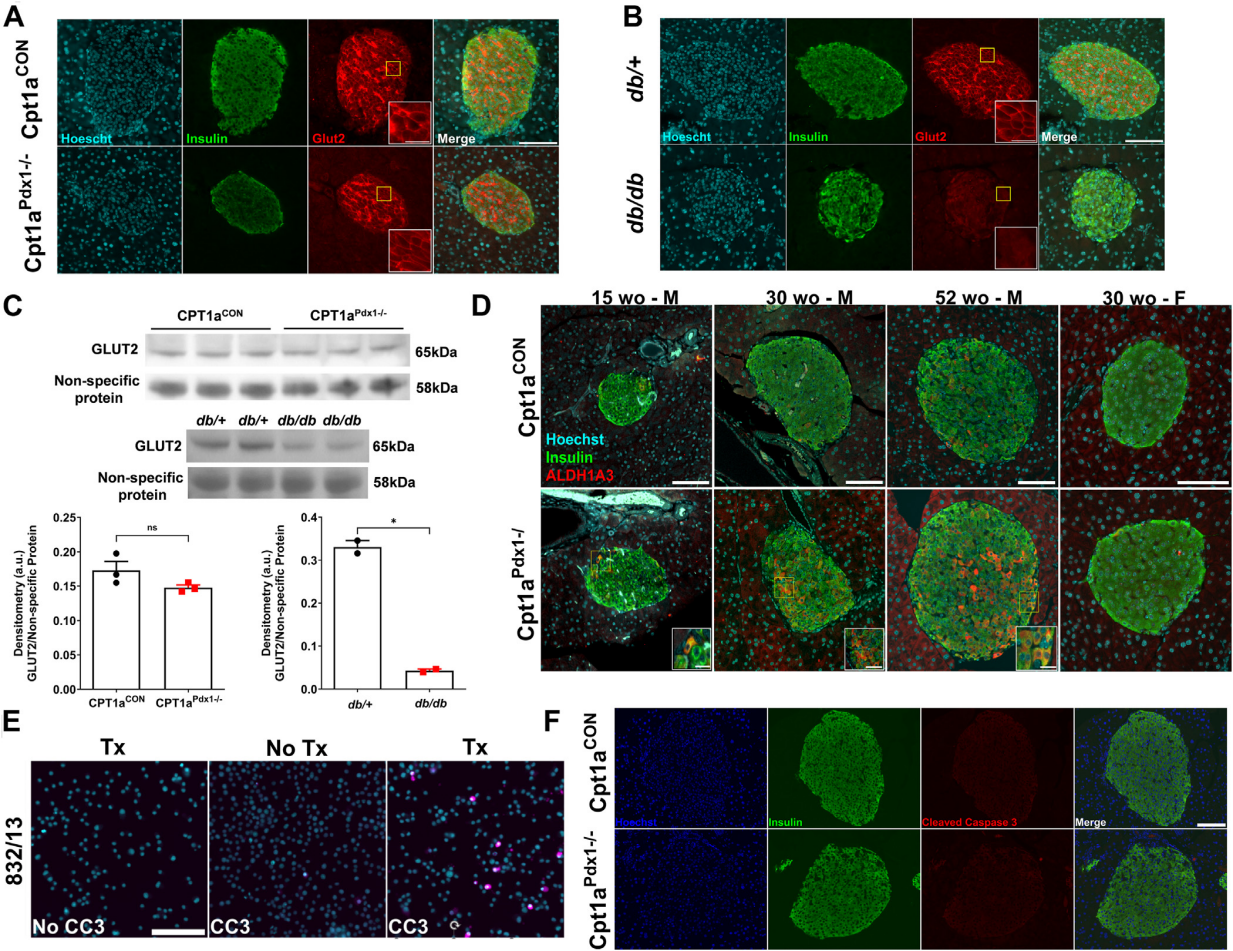
**GLUT2 abundance is unchanged, but the dedifferentiation marker, ALDH1A3, is elevated in β-cells from male, but not female, micedeficient in CPT1a.***A*, immunofluorescence staining for insulin (*green*) costaining with GLUT2 in *red* in FFPE tissue from 30 week old male Cpt1a^CON^*versus* Cpt1a^Pdx1−/−^ mice and (*B*) 30 week old male *db/+ versus db/db* mice. *C*, GLUT2 abundance was determined in islets isolated from Cpt1a^CON^*versus* Cpt1a^Pdx1−/−^ and *db/+ versus db/db* mice. Quantifications are shown underneath immunoblots using a nonspecific protein detected by the GLUT2 antibody as the loading control. Islets from 2 to 3 mice were pooled and run in triplicate (*top panel*) or duplicate (*bottom panel*). *D*, immunofluorescence staining for insulin (*green*) costaining with ALDH1A3 (*red*) in FFPE tissue from 15, 30, and 52 week old male and 30 week old female Cpt1a^CON^*versus* Cpt1a^Pdx1−/−^ mice. *E*, 832/13 insulinoma cells either untreated (No Tx) or treated for 8 h with 2 μM camptothecin (Tx). Cells were fixed in 2% formaldehyde followed by immunofluorescence staining for cleaved caspase 3 (CC3). *F*, immunofluorescence staining for insulin (*green*) and cleaved caspase 3 (*red*) in FFPE tissue from 52 week old male Cpt1a^CON^*versus* Cpt1a^Pdx1−/−^ mice. n = 5 per genotype (A-B and D, F). Scale bars represent 100 μm, inset scale bar represents 20 μm. M = male and F = female. ns = not significant, ∗*p* < 0.05. CPT1A, carnitine palmitoyltransferase 1A; FFPE, formalin-fixed, paraffin-embedded; GLUT2, glucose transporter 2.

Given the trend toward a decrease in *Slc2a2* transcript levels (Fig. 6*A*), we examined GLUT2 protein levels and found that colocalization of GLUT2 with insulin-positive β-cells is similar between genotypes (Fig. 7*A*). This is in contrast with the decline in GLUT2 observed in islets of models of T2D, for example, *db/db* mice (Fig. 7*B*) and ZDF rats (14). These findings were confirmed by immunoblot analysis (Fig. 7*C*).

ALDH1A3 has been identified as a marker of β-cell dedifferentiation in human (15, 16) and mouse models of T2D (17, 18) and has also been detected in other models of β-cell dysfunction (19, 20, 21). Very few ALDH1A3-positive β-cells were detected in islets of littermate control Cpt1a^CON^ male mice at any age (Fig. 7*D*). The presence of ALDH1A3 was observed as early as 15 weeks of age in male Cpt1a^Pdx1−/−^ mice (Fig. 7*D*). The population of ALDH1A3-positive islets drastically increased from 15 weeks of age to 1 year of age in male mice. In addition, ALDH1A3 was detected in exocrine tissue beginning at 30 weeks of age and increasing in intensity at 1 year of age in male Cpt1a^Pdx1−/−^ mice (Fig. 7*D*). In contrast, very few ALDH1A3-positive β-cells were noted in female Cpt1a^Pdx1−/−^ mice at 30 weeks of age, with no detection of ALDH1A3 in the exocrine compartment (Fig. 7*D*). Therefore, ALDH1A3 positivity is present in male mice with *Cpt1a* deletion in pancreatic tissue, but this phenotype is minimal in female mice.

To determine whether the decrease in insulin positive area (Fig. 3*I*) and β-cell mass (Fig. 3*K*) observed in islets of 1 year old male Cpt1a^Pdx1−/−^ mice could also be due to increased apoptosis, we examined cleaved caspase 3. As a positive control, we showed increased abundance of cleaved caspase 3 by immunofluorescence (IF) staining in 832/13 rat insulinoma cells treated with the apoptotic inducer camptothecin (22) (Fig. 7*E*) coinciding with a 40% loss in cellular viability assessed by MTS assay (data not shown). Using similar staining conditions, it is evident that pancreatic deletion of CPT1A does not elicit β-cell apoptosis (Fig. 7*F*).

### Minimal changes in global transcripts or mitochondrial content, but reduced ATP production, is observed in male Cpt1a^Pdx1−/−^ mice

**Figure 8:**
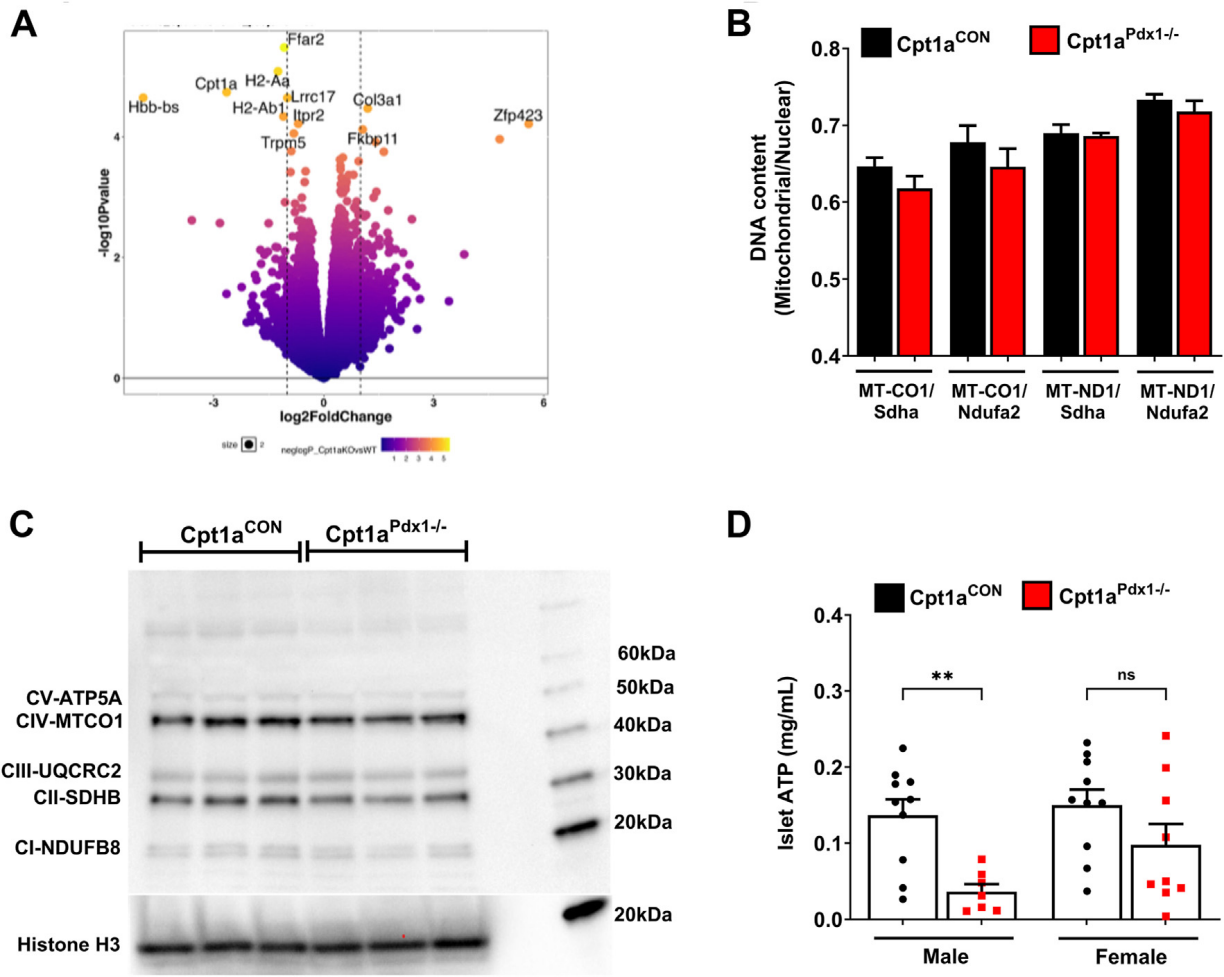
**Minimal changes in global transcripts or mitochondrial content, but reduced ATP production, is observed in male Cpt1a**^**Pdx1−/−**^**mice.***A*, volcano plot showing differentially expressed genes in isolated islets from male Cpt1a^CON^*versus* Cpt1a^Pdx1−/−^ mice, n = 3. *B*, mitochondrial number was calculated using the ratio of mitochondrial DNA (MT-CO1 or MT-ND1) relative to nuclear DNA (Sdha or Ndufa2) in genomic DNA isolated from male Cpt1a^CON^*versus* Cpt1a^Pdx1−/−^ mice, n = 8. *C*, immunoblot analysis of total OXPHOS in isolated islets from male Cpt1a^CON^*versus* Cpt1a^Pdx1−/−^ mice. Each band represents islets pooled from two animals. Blot repeated on three separate occasions. *D*, intracellular measurement of ATP levels using 50 islets from male and female Cpt1a^CON^*versus* Cpt1a^Pdx1−/−^ mice between 15 and 30 weeks of age, n = 7 to 10. ns = not significant, ∗∗*p* < 0.01. CPT1A, carnitine palmitoyltransferase 1A.

RNA-Seq revealed that very few transcripts were altered in the islets of Cpt1a^Pdx1−/−^ relative to littermate control mice (Fig. 8*A*). We interpret this data to indicate that the responses conferred by pancreatic CPT1A deletion are largely metabolic. In addition, the ratio of mitochondrial to nuclear DNA was not different (Fig. 8*B*), and the presence of proteins of the electron transport chain were not changed between the genotypes (Fig. 8*C*). By contrast, intracellular ATP quantities were reduced in male, but not female, Cpt1a^Pdx1−/−^ mice relative to littermate controls (Fig. 8*D*). Thus, we conclude that in male mice, reducing pancreatic CPT1a activity limits ATP production without major changes in global transcripts or markers of mitochondrial content. These are also consistent with female mice, which have largely normal insulin secretion responses, as they maintain intracellular ATP levels in the face of pancreatic CPT1A deletion.

## Discussion

Intracellular lipid is required to support physiological GSIS (2). An acute increase in fatty acid availability can potentiate GSIS; however, chronic ectopic lipid storage in pancreatic tissue impairs β-cell function and is associated with T2D in humans and rodents (23, 24, 25). When lipid accumulates in a tissue, there are routes to accommodate the excess, for example, sequestration of TGs in lipid droplets or catabolism through fatty acid oxidation. Islets can adapt to the accumulation of excess lipids during overnutrition with a compensatory enhancement of fatty acid oxidation. For example, Sprague Dawley rats fed a 60% fat diet for 70 days (*versus* a 4% fat diet) showed a 30% increase in palmitate oxidation in the islet (26). Similarly, exposure of human islets to fatty acids for 48 h also resulted in a 30% increase in fatty acid oxidation (27). In islets isolated from leptin-resistant ZDF *fa/fa* rats, palmitate oxidation is decreased by 77% compared to nondiabetic lean control *fa/+* rats (28), consistent the lipid accrual in this model of obesity and T2D. This suggests an eventual loss of this compensatory protective mechanism in obese, diabetic, or leptin resistant animals, producing an overwhelming lipid burden on the β-cells: indeed, the TG content in the islets of ZDF rats are more than 75% higher than islets from Sprague Dawley rats fed a 60% fat diet (29).

Islet β-cell insulin secretion is a tightly controlled process that consists of multiple steps, which are often referred to as stimulus-secretion coupling. Several reviews have discussed in depth the metabolism of glucose and its link to triggering the release of packaged insulin granules (30, 31). In addition, there were excellent past studies that documented the requirement for circulating tissue fatty acids to support insulin secretion (2, 3). However, whether islet fatty acid oxidation directly participates in GSIS and helps to maintain glucose homeostasis *in vivo* is incompletely understood. Previous examination of the role of mitochondrial fatty acid oxidation on GSIS *in vitro* has suggested that increasing fat oxidation reduces GSIS (32), while inhibiting fat oxidation improves GSIS (6, 7, 8, 33, 34, 35). However, a key limitation of these studies is they exclusively used *in vitro* model systems including β-cell lines, isolated pancreatic islets, or isolated pancreas tissue exposed to pharmacologic inhibitors or other manipulations of the enzyme CPT1 (6, 7, 8, 32). The *in vivo* pursuit of mechanisms to explain these intriguing early results depended on the generation of a novel genetic model, whereby fatty acid entry into mitochondria could be limited. Toward this goal, we developed, to our knowledge, the first genetic model with pancreatic deletion of CPT1A. The advantage of this model system is that the *Pdx1* gene is one of the early-expressed genes during pancreas development and one that persists through β-cell maturation. Therefore, the Pdx1 promoter driving Cre expression is robustly expressed in β-cells with a lower level of expression in acinar cells and other endocrine cells (36, 37).

CPT1 is a mitochondrial enzyme that catalyzes the formation of long-chain acyl-carnitine from long-chain acyl-coenzyme A. CPT1A is rate limiting for fatty acid transport into the mitochondria in the β-cell, and therefore plays a critical role in partitioning of fatty acid metabolism toward mitochondrial oxidation, thus helping with physiological distribution of long-chain fatty acids into oxidation *versus* storage in the form of TGs. The *Cpt1a* gene has been shown to be upregulated transcriptionally by palmitate and oleate in INS-1 cells (38) and similarly by incubation of human islets from nondiabetic donors with oleate (27); therefore, increased abundance of CPT1 may facilitate increased fatty acid oxidation in the face of excess intracellular lipid. Once inside the mitochondria, fatty acids are metabolized through the beta oxidation pathway to generate ATP. The generation of ATP is critical for closure of ATP-gated potassium channels. This rise in ATP after a glucose stimulation historically has been referred to as part of the mechanism regulating “first phase” insulin secretion (30, 39), which is also called the “triggering pathway” (31). Since ATP is a critical regulatory component for various aspects of islet β-cell function, we reasoned that reduction in islet fatty acid metabolism would reveal its contribution to insulin secretory outcomes by potentially lowering the total pool of ATP to serve as a secretory coupling signal.

Our novel data using male Cpt1a^Pdx1−/−^ mice clearly revealed that limiting mitochondrial lipid entry promotes tissue accumulation of intracellular lipids (Fig. 1, *D* and *E*), with increased intensity of Raman peaks in islets of Cpt1a^Pdx1−/−^ mice indicative of elevated levels of TGs, and also possibly increased phospholipids and saturated fatty acids (Figs. 1*E*, S1 and S2). Accumulation of lipids in pancreatic tissue and islets of Cpt1a^Pdx1−/−^ mice correlates with an impairment in glucose tolerance (Fig. 3, *A*–*D*) without any alteration in body composition or insulin sensitivity (Fig. 1). This reduction in glucose tolerance coincided with a decrease in glucose- and KCl-induced insulin secretion (Fig. 3, *E*–*G*). Furthermore, we demonstrated a clear requirement for CPT1A-dependent mitochondrial lipid entry for lipid potentiation of GSIS *in vivo* (Fig. 4, *C*–*F*). These data are consistent with McGarry’s model of *in vivo* lipid requirement for insulin secretion (2) and provide a mechanistic explanation for those earlier findings. CPT1A-deficient mice also display an impairment in whole body lipid clearance (Fig. 4, *A* and *B*), suggesting that pancreatic CPT1-A assists with clearance of lipid from circulation. Additionally, the impairment of GSIS is not impacted by dietary fat intake (Fig. 4, *M* and *P*); therefore, we conclude that the primary factor impairing GSIS is loss of CPT1A abundance.

As a possible explanation for the decrease in insulin output in CPT1A-deficient mice, we analyzed islet β-cell maturity markers and found no differences in Nkx6.1, MafA, or Pdx1 (Fig. 6, *A*–*C*). GLUT2 is reduced in *db/db* mice (Fig. 7, *B* and *C*) and in islets of Zucker diabetic fatty rats (14), which are rodent models displaying increased tissue lipid accrual associated with glucose intolerance and frank diabetes. We note that there is a 45.7% reduction in *Slc2a2* mRNA in Cpt1a^Pdx1−/−^ relative to littermate control mice (Fig. 6*A*), but no obvious difference in cellular membrane-associated GLUT2 (Fig. 7, *A*–*C*).

By contrast, ALDH1A3 was elevated in the islets of male mice with pancreatic CPT1A deletion (Fig. 7*D*). The observed decrease in insulin-positive area (Fig. 3*I*), islet fraction (Fig. 3*J*), and β-cell mass (Fig. 3*K*) at 52 weeks of age in male Cpt1a^Pdx1−/−^ mice, despite no change in pancreas mass (Fig. 3*L*), may be due to the gradual increase with age in β-cell dedifferentiation (Fig. 7*D*), with the possibility that maturity markers do not decrease in abundance in remaining β-cells. Further, we found no evidence of β-cell death by apoptosis in islets of older Cpt1a^Pdx1−/−^mice (Fig. 7*F*). Despite these alterations, circulating insulin levels remain unchanged (albeit trending lower) between genotypes at 1 year of age (Fig. 6*D*).

RNA-Seq did not reveal gross changes in transcriptional reprogramming suggesting that genetic reductions in *Cpt1a* largely produced metabolic phenotypes (Fig. 8*A*). Consistent with this notion, we observed that, under normal conditions, islet ATP levels were markedly reduced in male mice with pancreatic CPT1A deficiency (Fig. 8*D*). We note that a 64% decrease in CPT1 enzymatic activity (Fig. 1*C*) correlated with a 73% decrease in intracellular ATP content (Fig. 8*D*). Considering that completion oxidation of glucose produces approximately 30 ATP (40) *versus* 106 ATP for oxidation of palmitate (40), it is not surprising that inability to metabolize long-chain fatty acids, such as palmitate, lead to a global lowering of the intracellular ATP levels. ATP production from glucose, amino acids, and medium to short-chain lipids would not be altered, thus providing sufficient ATP for cellular viability but not to fully support physiological insulin secretion. Thus, our studies using novel genetic models have revealed a previously suspected, but unproven, role for fatty acids to support insulin secretion. Similar to our findings showing that pancreatic CPT1A deletion lowers intracellular ATP levels in islets, evidence suggests that maintenance of glucagon secretion in α-cells also relies on ATP generated from fatty acid oxidation rather than glucose induced increases in ATP (12).

In contrast to male mice, female Cpt1a^Pdx1−/−^ mice do not display a β-cell phenotype despite an increase in pancreatic lipid accumulation (Fig. 2*B*) and mild glucose intolerance (Fig. 5*B*). Glucose- and KCl-induced insulin secretion (Fig. 5, *C* and *D*) trends lower in isolated islets from female Cpt1a^Pdx1−/−^ mice but does not reach statistical significance between genotypes (Fig. 5, *C* and *D*). Furthermore, no detectable levels of ALDH1A3 are observed in female Cpt1a^Pdx1−/−^ mice (Fig. 7*D*) and female mice do not display reduced intracellular ATP levels (Fig. 8*D*) and thus maintain physiological insulin secretion. We interpret this data to indicate a sex specific differential usage in substrates (amino acids, medium-chain fatty acids, *etc.*) to support insulin secretion. Whether there is compensation from other CPT isoforms in the β-cell of female mice also remains undetermined. Future studies will address this interesting observation.

The novel data described in this study is distinct from the studies where CPT1A activity has been modulated entirely in cell culture or *in vitro* conditions. For example, inhibition of CPT1A with etomoxir produced an increase in insulin secretion in insulinoma cells (6, 7, 8), and conversely, when CPT1A was overexpressed in insulinoma cells, GSIS was decreased (32). There are at least two possible explanations for these differences. The first is that modulating CPT1A activity within pancreatic tissue *in vivo* could have different consequences than manipulations conducted using β-cell lines. Another possibility is that exocrine/endocrine cell crosstalk is an important factor regulating islet β-cell function. Indeed, increased pancreatic lipid deposition has been postulated as the single most important risk factor for diseases of both the exocrine and endocrine pancreas (41, 42).

We acknowledge several limitations within the current study. First, while the Pdx1-Cre is a powerful driver of Cre expression in β-cells, this model has the disadvantage of being detected in the hypothalamus, and in other endocrine cells. Some Pdx1-Cre driver lines have also been shown to cause recombination in duodenum, antral stomach, bile duct, and inner ear (36, 37). Whether recombination in nonpancreatic tissue or other endocrine cells of the islet is partially responsible for the phenotypes observed therefore cannot be determined, for example, α or δ cells are known to elicit paracrine signals that may regulate β-cell function (43). However, knockout of CPT1A specifically in α-cells showed that while CPT1A is essential for glucagon secretion, it had no impact of insulin secretion (12). Second, given the contradictory findings of previous *in vitro* studies using insulinoma cells lines with the *in vivo* and *ex vivo* outcomes obtained in the current study, no *in vitro* investigation was performed in the current study. Generation of additional genetic models in future studies, for example, using Ins1-Cre will allow us to further probe the mechanism responsible for decreased mitochondrial function and β-cell output. Finally, evaluation of exocrine pancreatic function was not performed as our primary goal was to evaluate endocrine function.

In summary, we have identified that pancreatic CPT1A activity is critical for maintenance of whole body glucose tolerance. Reductions in the ability to effectively metabolize long-chain fatty acids in pancreatic tissue could represent an early defect in pancreatic islets that leads to tissue lipid accumulation (Fig. 1, *D* and *E*), presence of ALDH1A3 (Fig. 7*D*), and contributes to the observed reductions in GSIS (Fig. 3, *E*–*G*) and β-cell mass (Fig. 3*K*). The decrease in insulin secretion can be attributed, at least in part, to a decline in intracellular ATP levels in the islet (Fig. 8*D*). Our data are therefore consistent with existing data showing that the presence of lipids are necessary to sustain GSIS (2) and provide new insights by showing that the pancreatic CPT1A pathway is an important component of regulated insulin secretion and glucose homeostasis *in vivo*. Given the adaptive response in fatty acid oxidation in rodent models exposed to excess lipid, the question remains whether overexpression of CPT1A *in vivo* can provide a route for metabolism of excess lipid and prevent impairments in endocrine function in models of obesity and T2D. Such studies await the development of novel genetic models.

## Experimental procedures

### Cell culture and reagents

832/13 rat insulinoma cells (44) were cultured as described previously (22). Cells were free from *mycoplasma* contamination for all experiments. For measurements of cell viability, 832/13 cells were treated with 2 μM camptothecin (MilliporeSigma) for 8 h and viability was measured by CellTiter 96 AQ_ueous_ One Solution Cell Proliferation MTS Assay (Promega) similarly to a previously published experiment (22). A parallel-treated plate was fixed with 2% formaldehyde (MilliporeSigma) and IF staining for cleaved caspase 3 was performed (described below in “Cell Line and Pancreas Histology”).

### Mouse models and islet isolation

All studies were performed in accordance with the guidelines of the NIH and were approved by the Pennington Biomedical Research Center Institutional Animal Care and Use Committee. *db/+* and *db/db* mice were from The Jackson Laboratory (Stock # 000697). Mice with a pancreas-specific deletion of *Cpt1a* (Cpt1a^Pdx1−/−^) were generated by crossing Cpt1a^fl/fl^ mice (C57BL/6-*Cpt1a*^*tm1.1mrl*^ Model # 9759 from Taconic) with Pdx1-Cre mice (Stock # 014647; Jackson Laboratory, Bar Harbor, ME). The control group (Cpt1a^CON^) was comprised of three genotypes: nonfloxed, Cre-negative; fl/fl, Cre-negative; and nonfloxed Cre-positive mice; no difference was observed across these three genotypes for any parameter examined. Mice were group-housed with a 12-h light/12-h dark cycle in a temperature- (22 ± 2 °C) and humidity-controlled room with *ad libitum* access to water and Rodent Diet 5015 (26% fat kcal; LabDiet). Multiple cohorts of mice were required for completion of the studies described with littermate controls used for all experiments (individual animal numbers for each experiment are provided in the respective figure legends). Islet isolation was performed using our previously published protocol (45, 46). Briefly, islets were isolated by enzymatic digestion using Liberase TL (MilliporeSigma), followed by separation of islets by sucrose gradient using Polysucrose Ficoll 400 (MP Biomedicals) and isolation by handpicking.

### Blood glucose, body composition, and metabolic cage analysis

Measurements of random-fed blood glucose were taken every 4 weeks using a Bayer Contour glucometer. Body mass and composition (fat, lean, and fluid mass) was assessed every 4 weeks starting when mice were 6 weeks of age by NMR spectroscopy using a Bruker Minispec LF110 Time-Domain NMR system. Indirect calorimetry was performed on mice at 12 weeks of age as previously described by our group (18, 46) using Promethion metabolic cages (Sable Systems).

### Mitochondrial preparations and CPT1 activity assay

Preparation of mitochondrial suspensions from pancreatic tissue and determination of CPT1 enzymatic activity were as described previously (47). Briefly, CPT1 activity was determined using 10 μl of the intact pancreatic mitochondrial suspension (0.2–0.4 mg mitochondrial protein per reaction). The concentration of protein in mitochondrial suspensions was quantified by bicinchoninic acid protein assay (Thermo Fisher Scientific). Mitochondria were incubated in the presence of 50 μM palmitoyl-CoA and [^3^H]L-carnitine. Reactions were stopped following a 6 min reaction at 37 °C and [^3^H]palmitoyl-carnitine was extracted with water-saturated butanol and quantified by liquid-scintillation counting. Assays were performed in triplicate and results were normalized to mitochondrial protein content.

### Insulin tolerance test and serum ELISAs

Insulin sensitivity was assessed by insulin tolerance test following a 2 h fast. Mice were injected into the peritoneal cavity with 1 U/kg lean mass of insulin (Humulin R). Blood glucose measurements were taken at 0, 15, 30, 45, 60, and 90 min. Serum insulin (Cat # 10-1247-10), proinsulin (Cat # 10–1232–01), and glucagon (Cat # 10-1281-01) were measured using ELISA kits from Mercodia according to the manufacturer’s instructions. Mouse C-peptide was quantified using an ELISA kit from ALPCO (Cat # 80-PTMS-E01).

### Glucose tolerance tests, *ex vivo* glucose-stimulated insulin and glucagon secretion

For measurements of glucose homeostasis, male (12, 24, and 52 weeks of age) and female (16–28 weeks of age) mice underwent glucose tolerance tests (GTTs). Following a 4 h fast, animals received an intraperitoneal injection of 2.5 g of glucose/kg of body weight. Blood glucose levels were measured from tail vein blood at 0, 20, 40, 60, and 120 min *via* a Bayer Contour glucometer. Perifusion analyses of insulin secretion and quantification of insulin content were performed by the Islet Procurement and Analysis Core at Vanderbilt University using isolated islets from both male and female Cpt1a^CON^ and Cpt1a^Pdx1−/−^ mice. Data were normalized to 100 islet equivalents. Measurements of glucagon secretion were performed on 100 islet equivalents from 12 week old male Cpt1a^CON^ and Cpt1a^Pdx1−/−^ mice. Briefly, islets were exposed to either 5.6 mM or 16.7 mM glucose in Dulbecco's modified Eagle's medium–based media for 1 h. Glucagon secretion into the buffer was measured using the Mercodia Glucagon ELISA kit.

### Mouse intervention studies

For measurements of β-cell proliferative capacity (Fig. 3, *M* and *N*), Cpt1a^CON^ and Cpt1a^Pdx1−/−^ mice were placed on either a 10% kcal from fat (Cat #D12450J; Research Diets) or 45% kcal from fat (Cat #D12492; Research Diets) diet for 1 week. A separate cohort was given an oral corticosterone regimen (100 μg/ml in 1% EtOH; Cat # 27840, MilliporeSigma) or vehicle control (1% EtOH) *via* their drinking water for 1 week using previously described methods (9, 48).

Whole body lipid tolerance was assessed by lipid tolerance test following a 16 h fast. Fifteen week old male mice were gavaged with 20% Intralipid emulsion (MilliporeSigma; Cat #I141-100 ML). Blood was collected *via* the tail vein at 0, 1, 2, 3, 4, 5, 6, and 8 h. Blood was centrifuged at 7200*g* for 20 min and the serum fraction used for determination of TG content using the Serum TG Determination Kit from MilliporeSigma (Cat # TR0100; see next section).

For determination of GTT and GSIS with lipid, 15 week old male animals were fasted for 4 h. At −120 min, mice were gavaged with either saline or 20% Intralipid emulsion. At 0 min animals were then intraperitoneally injected with 2.5 g of glucose/kg of body weight. Blood glucose levels were measured from tail vein blood at −120, 0, 20, 40, 60, and 120 min *via* a Bayer Contour glucometer. Blood was collected *via* the tail vein at −120, 0, 20, and 60 min. Blood was centrifuged at 7200*g* for 20 min, and the serum insulin was calculated using the Mouse Insulin Kit from Mercodia.

For the dietary fat study (Fig. 4, *G*–*P*), a cohort of male 24 week old Cpt1a^CON^ and Cpt1a^Pdx1−/−^ mice were switched from Rodent Diet 5015 (26% fat kcal) to either Rodent Diet 5001 (13% fat kcal, *i.e.,* LFD) or modified Rodent Diet 5015 (44.3% fat kcal, *i.e.,* HFD). All diets used in these studies are from LabDiet with the modified Rodent Diet 5015 given the custom TestDiet catalog number 58QA. Body weight and blood glucose were monitored on a weekly basis, body composition and glucose tolerance were assessed at 0 and 8 weeks on diet by GTT, and *in vivo* GSIS was determined at 8 weeks on diet by collecting tail vein blood at 0 and 20 min time points during the abovementioned GTT.

Upon completion of each respective study, animals were fasted for 4 h and then euthanized by CO_2_ asphyxiation and decapitation. Trunk blood was collected for serum analyses. Pancreata were either snap-frozen in liquid nitrogen or fixed in 10% neutral-buffered formalin.

### Pancreatic TG determination

Pancreatic true TG (total TGs less free glycerol) was quantified from 50 mg of frozen, powdered pancreatic tissue from Cpt1a^CON^ and Cpt1a^Pdx1−/−^ mice using the Serum TG Determination Kit. First, samples were prepared by washing in cold PBS, followed by resuspension and homogenization in 5% NP-40 in ddH_2_0. Samples were then heated to 95 °C in a heat block until the NP-40 becomes cloudy then cooled to room temperature (RT) (repeated once more to completely solubilize all TG). Samples were centrifuged at 14,000*g* for 2 min to pellet insoluble material and TG content was quantified from the supernatant.

### Raman microspectroscopy

Islet TG scores were determined by Raman microspectroscopy using a Renishaw inVia Reflex Raman confocal microscope system for the spectra and imaging experiments. Ten islets each from 12 week old male Cpt1a^CON^ and Cpt1a^Pdx1−/−^ mice (n = 4 per genotype) were fixed in 3% paraformaldehyde and placed on stainless steel slides that were sterilized in 70% ethanol followed by washing with deionized water. For the Raman spectra acquisition, a 50X long-working distance objective lens, excitation laser wavelength of 785 nm, laser power of 9 mW, and acquisition time of 10 s were used. For all the imaging experiments, the same experimental parameters were used, except the acquisition time was 0.5 s. Glyceryl tripalmitate (Sigma-Aldrich, CAS # 555-44-2) was used as the Raman standard for TG in this work (TG (48:0)).

#### Raman data analysis

The preprocessing of Raman spectra was carried out as detailed in previous studies (49, 50, 51), involving the removal of cosmic ray artifacts and background subtraction. Principal component analysis (PCA) was employed to perform multivariate analysis on the Raman spectral data matrices, enabling visualization of cells exhibiting high spectral similarity (Fig. S1). For analysis, spectral data from four randomly selected cells were averaged, with at least 10 spectra obtained from each cell. Raman imaging of islet cells was conducted using a 68 × 54 raster scan over a 20 × 20 μm sample area, with a step size of 0.3 μm and an accumulation time of 0.5 s per step. In these imaging experiments, 6494 Raman spectra were collected from the Cpt1a^CON^ group and 3965 Raman spectra from the Cpt1a^Pdx1−/−^ group.

#### Direct classical least square analysis

Raman scores from the mapping images were determined using direct classical least square analysis. Direct classical least square is a supervised technique that uses Beer's Law (D = C∗S^T^ + E) to produce quantitative maps of the relative concentration (C) of components within a sample. S is the known component Raman spectra (S) or the standard. Here, E = error, and T = transpose of the matrix. The resulting images display the distribution of the components ranging from low to high concentrations. For example, if a Raman image has M × N pixels, each Raman spectrum with Q unique components (*e.g.*, protein, DNA, lipids, and collagen) and the dimensions of the matrix will be: C = M × Q, S = N × Q, and D = M × N.

### Cell line and pancreas histology

After fixation, cells were washed twice for 5 min with 1x tris-buffered saline + 0.1% Tween-20 (TBST), blocked for 1 h at RT with Blocker Casein in TBS (Thermo Fisher Scientific; Cat # 37583), and then incubated overnight at 4 °C with rabbit anti-Cleaved Caspase 3 (1:300; Cat # 9664; Cell Signaling Technology) or with antibody diluent only (negative control). The next day, the cells were washed with TBST three times for 5 min each and then incubated with Invitrogen Alexa Fluor Plus 488 Goat anti-Rabbit (Cat # A32731) at 1:500 in TBST for 2 h. The cells were then washed twice for 5 min each in TBST, stained with DRAQ5 (Cell Signaling Technology; Cat # 4084L), diluted 1:500 in TBST + sodium azide (0.05%), and then kept at 4 °C until imaging. Cells were imaged using wavelength appropriate filter sets on a Leica DMI6000B inverted microscope equipped with a long working distance 40x objective (Leica 11506203).

FFPE tissues were cut into 5 μm sections and placed on positively charged slides. Multiple sections were cut from each pancreata for downstream IHC and IF analyses. For determination of insulin-positive area, insulin-positive islets were detected using insulin staining from FFPE pancreatic tissue. These sections were analyzed and quantified using a custom application that detects insulin-positive area for each islet and islet fraction present within a section using the Visiopharm VIS software version 5.0.5 (www.visiopharm.com). For measurements of β-cell mass, the ratio of insulin-positive area to pancreatic area was multiplied by the pancreas wet weight. CPT1A was detected immunohistochemically by overnight incubation with a Mouse anti-Cpt1a antibody (Cat # ab128568; Abcam). Tissues were then incubated with Goat anti-Mouse IgG2b horseradish peroxidase (HRP) at 1:300 (Jackson Immunoresearch; Cat # 115-035-207), followed by staining with the ImmPACT DAB substrate Kit, peroxidase (HRP) from Vector Laboratories (Cat # SK-4105). For IF staining, the following primary antibodies were used: Guinea Pig anti-insulin (Abcam Cat # ab7842 1:500), Rabbit anti-MafA (Bethyl Laboratories Cat # IHC-00352 1:50), Mouse anti-Nkx6.1 (Developmental Studies Hybridoma Bank Cat #F55A10 1:200), Rabbit Anti-GLUT2 (Proteintech Cat # 20436-1-AP 1:500), Mouse anti-Glucagon (eBioscience Cat # 14-9743-82 1:400), Rabbit Anti-ALDH1A3 (Novus Bio Cat # NBP2-15339 1:200), Rabbit anti-Ki67 (Fortis Life Sciences Cat # IHC-00375 1:300), and Rabbit anti-cleaved caspase 3 (Cell Signaling Technology Cat # 9664 1:300). FFPE sections were dewaxed and subjected to heat-induced epitope retrieval (20 min at 100 °C, Biocare Medical Decloaking Chamber) with either a pH 6.0 citrate [ALDH1A3, glucagon, Ki-67] or pH 9.0 tris-based buffer [MafA, Nkx6.1] (H-3300, H-3301; Vector Laboratories). Prior to blocking with casein (Cat # 37583; Thermo Fisher Scientific) and incubation with primary antibodies overnight at 4 °C, slides to be stained for MafA and Nkx6.1 were incubated with a 3% H_2_O_2_/TBST solution for 10 min at RT to block endogenous peroxidases. After washing slides 3 times in TBST, ALDH1A3, insulin, glucagon, and Ki-67 were detected *via* an 1 h incubation with fluorescently conjugated secondary antibodies at 1:300 (A11073-Goat anti-Guinea Pig Alexa488, A11037-Goat anti-Rabbit Alexa594; Invitrogen). MafA and Nkx6.1 were detected by first incubating slides with a goat anti-rabbit HRP (Leica Biosystems) or goat anti-Mouse IgG1 subclass specific HRP-conjugated antibody (115-035-205; Jackson Immunoresearch) for 30 min. After washing in TBST 3 times, the slides were then incubated with PerkinElmer Cy3-TSA (1/100 in amplification buffer) for 10 min. All slides were washed in TBST prior to counterstaining with Hoechst and mounting in Vectashield Vibrance (H-1700; Vector Laboratories). Whole slide scans were collected on a Hamamatsu NanoZoomer HT equipped with a 20x/0.8 NA objective. Images of individual islets were captured on a Leica DM6000 B with a 40x/0.95 NA air objective and Leica K5 sCMOS camera.

### RNA isolation, complementary DNA synthesis, and RT-PCR

Total RNA was isolated from Cpt1a^CON^ and Cpt1a^Pdx−/−^ mouse islets using the RNeasy Mini RNA kit from Qiagen. Procedures for complementary DNA synthesis, primer design, and RT-PCR were performed using our standard laboratory protocol (13).

### Immunoblotting

For detection of proteins by immunoblotting islets from *db/+*, *db/db*, Cpt1a^CON^, and Cpt1a^Pdx−/−^ mice were isolated and islets from each genotype were pooled (n = 2–3 mice per genotype with approximately 100 islets per mouse) and run in duplicate or triplicate. Islet lysis, protein quantification, and immunoblotting methods are as described (46, 48). The following primary antibodies were used: rabbit anti-GLUT2 from Proteintech (1:1000; Cat # 20436-1-AP), mouse anti-Total OXPHOS from Abcam (1:1000; Cat #ab110413), and Histone H3 from Cell Signaling Technology (1:1000; Cat # 3638). The GLUT2 antibody detects a nonspecific protein at 58 kDa which was used as the loading control in Figure 7*C*. Secondary antibodies were from Cell Signaling Technology: HRP-linked anti-rabbit IgG (1:5000; Cat # 7074S) and HRP-linked anti-mouse IgG (1:5000; Cat # 7076S).

### Serial analysis of gene expression and gene expression data analysis

For RNA-seq analysis, RNA was isolated from 24 week old male Cpt1a^CON^ and Cpt1a^Pdx−/−^ mouse islets. Sequencing libraries were constructed using a Quant-Seq 3′ mRNA-Seq Library Prep kit (Lexogen SKU: 015.96) following the standard protocol for low input samples. Each library was constructed using unique library indexes. Completed libraries were analyzed on the Bioanalyzer High Sensitivity DNA chip (Agilent) to verify correct library size. All libraries were pooled in equimolar amounts and sequenced on the NextSeq 500 sequencer (Illumina) at 75 bp forward read and 6 bp forward index read. Primary data analysis was performed using the Lexogen Quantseq pipeline V1.8.8 on the BlueBee platform. Four biological replicates per genotype from male mice were submitted for sequencing. The raw and processed data are deposited in the gene expression omnibus (GEO) database (accession number GEO: GSE262423).

#### Differential gene expression analysis

Raw count matrices of RNA-seq data were obtained *via* the Rsubread (52) package in R, and further normalized *via* the trimmed mean of M-values method (53), with normalization factors ranging from 0.96 to 1.06 for library sizes between 9.4 × 10^6^ and 1.35 × 10^7^ reads. Trimmed mean of M-value normalized counts were log2-transformed and used as input for PCA *via* the princomp package in R. PCA identified one Cpt1a^CON^ and one Cpt1a^Pdx−/−^ samples as outliers which were removed from subsequent analysis. After outlier removal, genes with at least one count per million (CPM) reads in three or more samples were retained for further analysis, resulting in 12,943 genes. Differential gene expression analysis was conducted *via* the limma package in R (54), by first assessing the mean-variance relationship of gene-wise SD to average logCPM gene signal *via* the “voom” method (55) to generate precision weights for each individual observation. The logCPM values and associated precision weights were subsequently utilized to generate empirical Bayes moderated *t*-statistics estimates for the identification of differentially expressed genes. To adjust for multiple testing, genes with an adjusted *p* value <0.10 were considered to be differentially expressed.

### Mitochondrial/nuclear DNA ratio analysis, mitochondrial content, and ATP assay

Mitochondrial/nuclear DNA ratio analysis was performed as described previously (56). Intracellular ATP levels were determined from 50 islets per mouse per genotype, incubated in 11 mM glucose, using the ATP Bioluminescence Assay kit (MilliporeSigma Cat # 119107) according to manufacturer’s directions.

### Statistical analysis

Statistical analysis was performed using GraphPad Prism 10.1.1 (GraphPad Software; www.graphpad.com). All data were analyzed by two-tailed Student's *t* test, one-way ANOVA using a Tukey's *post hoc* or repeated measures ANOVA (for longitudinal measures of body weight and body composition, *etc.*). Datasets were tested for outliers using the Rout method (Q = 1%). Data are represented as means ± SEM.

## Data availability

RNA-Seq data are deposited in the publicly available GEO database (accession number: GSE262423).

## Supporting information

This article contains supporting information.

## Conflict of interest

The authors declare that they have no conflicts of interest with the contents of this article.

## Figures and Tables

**Supplementary Figure 2:**