# Supplementary material for: Pancreatic expression of CPT1A is essential for whole body glucose homeostasis by supporting glucose-stimulated insulin secretion
Source: J Biol Chem. 2025 Jan 13;301(2):108187. doi: 10.1016/j.jbc.2025.108187 (PMC11849070; doi:10.1016/j.jbc.2025.108187)
Supplement: Supplementary Figure Legends [file mmc1.docx]

**Supplementary Figure 1. Loss of CPT1A function in the pancreas of male mice reveals distinct Raman spectra and principal component analysis (PCA) in isolated islets.** (A) Raman spectra obtained from islet cells for the Cpt1a^CON^ (green) and Cpt1a^Pdx1-/-^ (brown) groups. The average spectrum is represented in dark colors, with the standard deviation shown as shaded regions. Both groups have *n* = 40. (B) Unsupervised PCA of the Raman spectra revealed distinct clustering between the Cpt1a^CON^ versus Cpt1a^Pdx1-/-^ groups. The first principal component (PC1) accounts for 55.1% of the variance, reflecting differences in the chemical signatures of the samples.

**Supplementary Figure 2. Raman spectra analysis in islets from male Cpt1a^CON^ versus Cpt1a^Pdx1-/-^ mice**. (A) Schematic illustrating the strategy for collecting multiple Raman spectra from a single islet cell. B) Representative Raman image of a single islet cell, captured at a resolution of 0.3 µm x 0.3 µm. Here, the Cpt1a^CON^ group image consists of 48 x 34 pixels, corresponding to *n* = 1,632 Raman spectra, while the Cpt1a^Pdx1-/-^ group image consists of 68 x 54 pixels, corresponding to *n* = 3,672 spectra. (C) Variable importance in projection (VIP) scores obtained from partial least-squares discriminant analysis (PLS-DA) of Raman peak intensities for the Cpt1a^CON^ and Cpt1a^Pdx1-/-^ groups. Blue squares indicate Raman peaks with lower intensities, while red squares denote peaks with higher intensities compared to the other group.
